# Low cost 3D printed clamps for external fixator for developing countries: a biomechanical study

**DOI:** 10.1186/s41205-020-00084-3

**Published:** 2020-10-23

**Authors:** Felix J. Landaeta, Jose Nauaki Shiozawa, Arthur Erdman, Cara Piazza

**Affiliations:** 1 Earl E. Bakken Medical Devices Center University of Minnesota-Twin Cities, 420 Delaware Street SE, MMC 95, G217 Mayo Building, Minneapolis, MN 55455 USA; 2 Centro de Salud B La Troncal, Instituto Ecuatoriano de Seguridad Social, Quito, Ecuador

**Keywords:** Linear external fixation, Biomechanical properties, Low cost, 3D print, Third world, Developing countries, ASTM F1541

## Abstract

**Background:**

External fixation is a mainstream limb reconstruction technique, most often used after a traumatic injury. Due to the high rates of trauma in developing countries, external fixation devices are often utilized for immediate fracture stabilization and soft tissue repair. Proper external fixation treatment too often still fails to be adopted in these regions due to the high cost and trauma complexity. A novel, inexpensive, unilateral fixator was constructed using 3D printed clamps and other readily available supporting components. ASTM standard F1541 tests were used to assess the biomechanical properties of this novel external fixator.

**Methods:**

Applicable sections of ASTM standard F1541 were used to determine the biomechanical properties of the novel external fixator. 3D printed clamps modeled using SolidWorks and printed with chopped carbon fibers using a fuse deposition modeling (FDM) based 3D printer by Markforged (Boston, MA) were used. This study included 3 different testing configurations: axial compression, anterior-posterior (AP) bending, and medial-lateral (ML) bending. Using the novel unilateral fixator with 3D printed clamps previously sterilized by autoclave, an input load was applied at a rate of 20 N/s, starting at 0 N via a hydraulic MTS tester Model 359. Force and deformation data were collected at a sampling rate of 30 Hz. There was a load limit of 750 N, or until there was a maximum vertical deformation of 6 mm. Also, 4 key dimensions of the 3D printed clamps were measured pre and post autoclave: diameter, width, height and length.

**Results:**

The novel external fixator had axial compression, AP and ML bending rigidities of 246.12 N/mm (σ = 8.87 N/mm), 35.98 N/mm (σ = 2.11 N/mm) and 39.60 N/mm (σ =2.60 N/mm), respectively. The 3D printed clamps shrunk unproportionally due to the autoclaving process, with the diameter, width, height and length dimensions shrinking by 2.6%, 0.2%, 1.7% and 0.3%, respectively.

**Conclusion:**

Overall, the biomechanical properties of the novel fixator with 3D printed clamps assessed in this study were comparable to external fixators that are currently being used in clinical settings. While the biomechanics were comparable, the low cost and readily available components of this design meets the need for low cost external fixators in developing countries that current clinical options could not satisfy. However, further verification and validation routines to determine efficacy and safety must be conducted before this novel fixator can be clinically deployed. Also, the material composition allowed for the clamps to maintain the appropriate shape with minimal dimensional shrinkage that can be accounted for in clamp design.

## Highlights

- 3D printed linear external fixator with ASTM F1541 compliant mechanical properties
- AO equivalent 3D printed linear external fixator
- Low cost 3D printed linear external fixator for the treatment of long-bone fractures

## Introduction

External fixation is the primary choice of temporary fracture stabilization for specific polytrauma patients [1]. Often, these traumatic injuries occur during motor accidents or are due to gun violence [2, 3]. The benefits of external fixation compared to other various bone fracture treatments is that it is less invasive, more versatile and does not induce as much swelling [4]. It has been shown that, if used properly, external fixators can treat bone and soft tissue pathologies with low risk and reasonably high success rates [2, 5]. The Arbeitsgemeinschaft fur Osteosysnthesefragen (AO) identifies external fixation as the first line of therapy for the treatment of AO-classified soft tissue injuries IC1 to IC4, as well as for the treatment of AO-classified long bone fractures AO32 (Femur) and AO42 (Tibia), among multiple other conditions [6].

External fixators are classified as Class II devices by the United States Food and Drug Administration. These fixators are normally provided worldwide by established medical technology companies such as Johnson and Johnson, Stryker and Orthofix. In countries such as the United States, with more complex healthcare systems, health insurance usually covers the cost of the fixator. However, in developing regions of the globe this is not the case. Therefore, cheaper, more universal unilateral external fixators are sought after in these regions. Often, the well-known AO generic external fixator currently attempts to meet the needs of developing countries, but proper external fixation treatment too often fails in these regions still, due to the high cost and trauma complexity [7].

Developing countries often utilize external fixation techniques due to a consistently high rate of trauma accidents that result in the need of bone fracture stabilization [8]. Current commercially available external fixation devices are complex and expensive, affecting management of injuries in less developed countries [9]. South East Asia and Africa, which comprise of low and middle-income countries, account for over 50% of the world’s traumatic injuries. Ultimately, there is a large disparity between regions that are affected by trauma and the resources that are available to treat such trauma [8].

There is a need for a low-cost, simple, readily available, safe and effective external fixation device that can be properly utilized by developing countries in order to treat the high rate of polytrauma patients with soft tissue injuries and long bone fractures. A low cost unilateral external fixation device that utilizes novel 3D printed clamps and other readily available parts was constructed to meet this need. The aim of the current study was to assess the biomechanical properties of this fixator and compare these properties to commonly used and commercially available fixators. Verification and validation routines dictated by global or local regulatory health agencies that are needed for a full clinical deployment of this fixator were not within the scope of this project.

## Materials and methods

### Assembly and set up of a linear external fixator

**Fig. 1:**
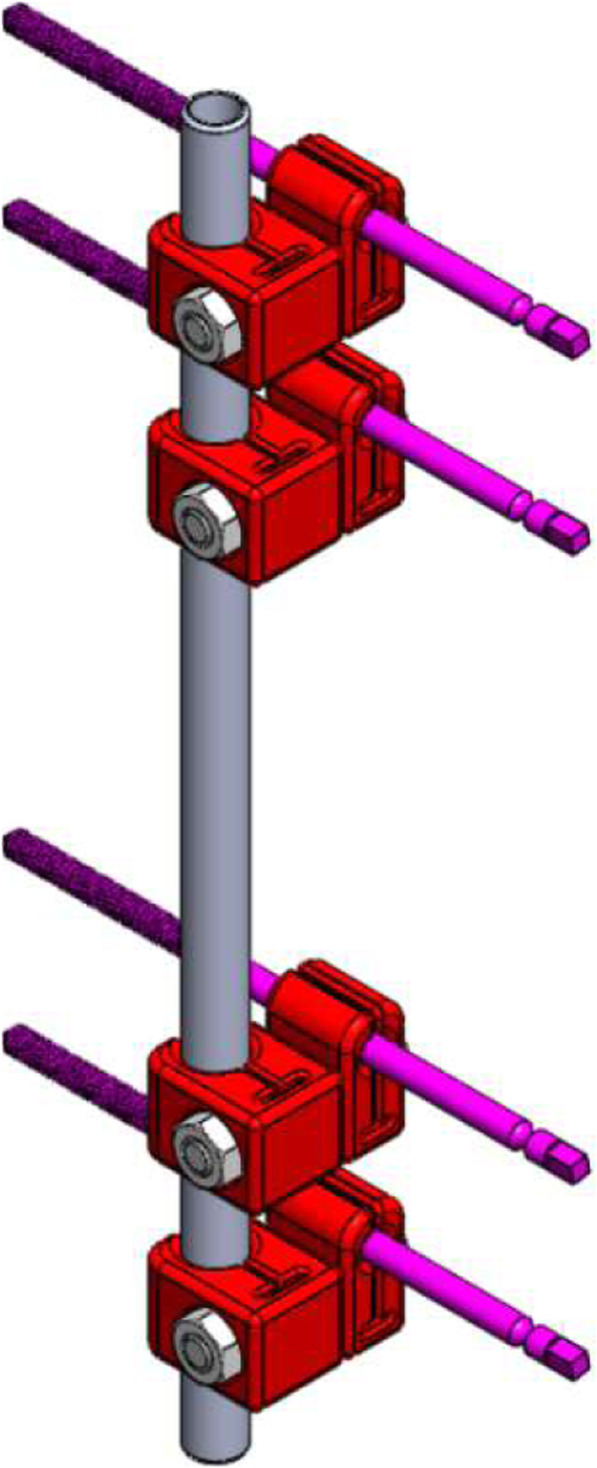
A computer model of the novel unilateral fixator. The red portions are the 3D printed parts

A rendering of the computer model frame assembly of the novel linear external fixator can be seen in Fig. 1. The single unilateral rod outside the plane of the bone screws is constructed by a grade 316 L stainless steel rod (300 m length, 12 mm diameter) with micro grooves to enhance friction. This type of rod is readily available at any material supplier store. To process this rod to the appropriate specifications, it was first cut via band saw to procure part length. Next, a 45 degree knurling die, which was 0.25 mm in depth, was used to procure the micro grooves via lathe knurling.

**Fig. 2:**
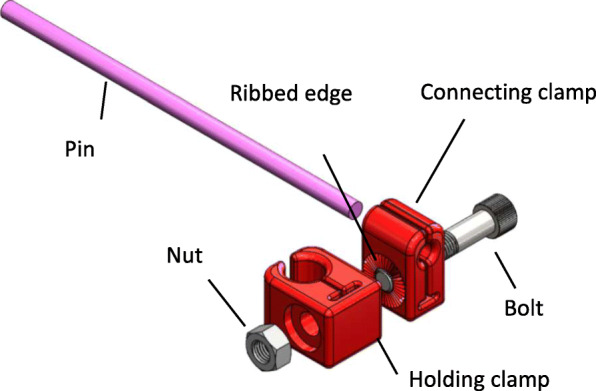
Two-part clamp which consists of the: 3D printed holding clamp, 3D printed connecting clamp, nut, bolt and pin. In this diagram, the Shanz screw is replaced by a pin, which was used in test set up

Four, two-piece 3D printed clamps sit on the axis of the rod. A clamp consists of two parts: the connecting clamp which holds a Schanz screw, and the holding clamp which is connected to the rod. The two-part 3D printed clamp is connected by nut and bolt (Fig. 2). The clamps were modeled using SolidWorks 2017 and 3D printed by a Markforged Onyx One (Boston, MA) printer using Fused Deposition Modeling (FDM) technique. In preparation for 3D printing, the models were converted into stereolithography (STL) format. The STL files were loaded into Markforged Eiger Printing software for parts slicing using the following parameters: layer height of 0.1 mm and 100% fill density. The material was Onyx by Markforged, which is nylon with chopped carbon fiber randomly distributed in the filament. This material is known to have a flexural strain larger than Acrylonitrile butadiene styrene’s (ABS), making it suitable for high strength demanding applications. In addition, Onyx is known to have a higher heat deflection temperature than Polylactic acid polymer’s (PLA) making it suitable for autoclave processing [10]. Nylon with chopped carbon fiber is available at any 3D print supplier store under other trade names. 3D parts were post processed in order to remove the supporting material defined for printing of the bolt hole. Supporting material was manually removed with no effects on part final geometry or surface finish.

The faces of the holding and connecting clamps are adjacent, and a ribbed design increases friction to hold the two-piece clamp together. The connecting clamp holds a Schanz screw of diameter 6 mm, made from stainless steel 316 L bars, commercially available, which sits perpendicular to the unilateral rod. For this study, the Schanz screw was substituted by a pin (6 mm in diameter) made also from stainless steel 316 L. A standard band saw was used to cut round bars to dimension to procure the pins. The material, length and diameter of these pins followed the standard specifications of Schanz screws commonly used in trauma clinical settings, the threaded portion was not manufactured.

### Test set up

The ASTM standard F1541 was used to guide the following test set up to determine the biomechanical properties of the novel fixator.

Prior to testing, a standard autoclave cycle was completed on the 3D printed holding and connecting clamps to assess the sterilizability by steam of these parts. The clamps were sterilized via autoclave in order to comply with ASTM standard F1541. Sterilirizability of the rod and pins was not evaluated since it is well known that stainless steel is an autoclave compatible product. The clamps were held at 135 °C for 25 min. Then, a process of cooling and drying brought the clamps back to room temperature over a span of 15 min. The autoclave used was a Sterilmatic STM-ED sterilizer, calibrated. Before autoclave and after autoclave parts were 100% visually inspected checking for surface finish defects. Likewise, before and after autoclave, general dimensions of the clamp were 100% measured using Mitutoyo Caliper Model CD-6"CSX, calibrated. The dimensions that were measured were the diameter where the rod interfaces with the clamp, clamp width, clamp height and clamp length. Control limits for dimensions were set at +/− 0.125 mm from nominal value.

**Fig. 3:**
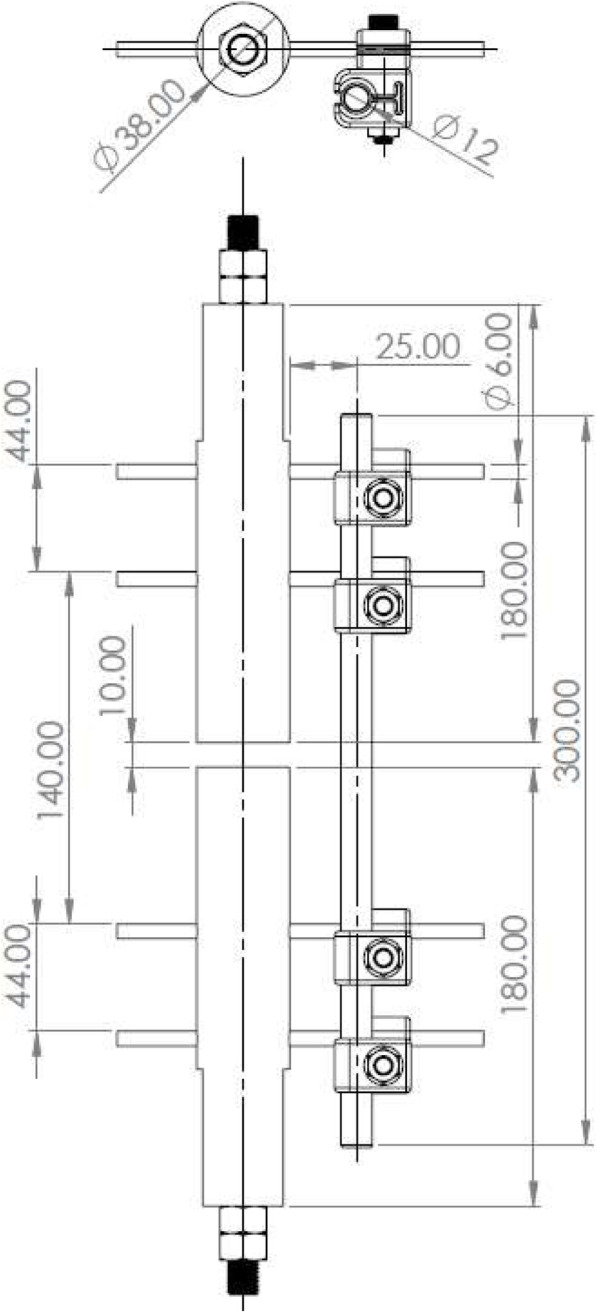
Schematic of the basic test set up. All dimensions noted are in millimeters

Ultra-high molecular weight polyethylene (UHWP) (38 mm diameter) was used as the synthetic bone substitute. The axis of two sections of UHWP rod were aligned and separated by a gap of 10 mm. The axis of the unilateral rod (12 mm diameter) was in the same vertical plane. The distance between the UHWP and rod axes was set at 44.05 mm. The holding clamps were aligned in this same plane. The connecting clamps were adjacent to the holding clamps and the rod. The axis of the column was 25 mm from the nearest side of the UHWP rod. A pin was placed in the holding clamp and inserted into the appropriate UHWP rod sections, pressed fit. The distance between each inter-pin was 44 mm and the distance between the innermost pins was 140 mm which can be seen in Fig. 3.

**Fig. 4:**
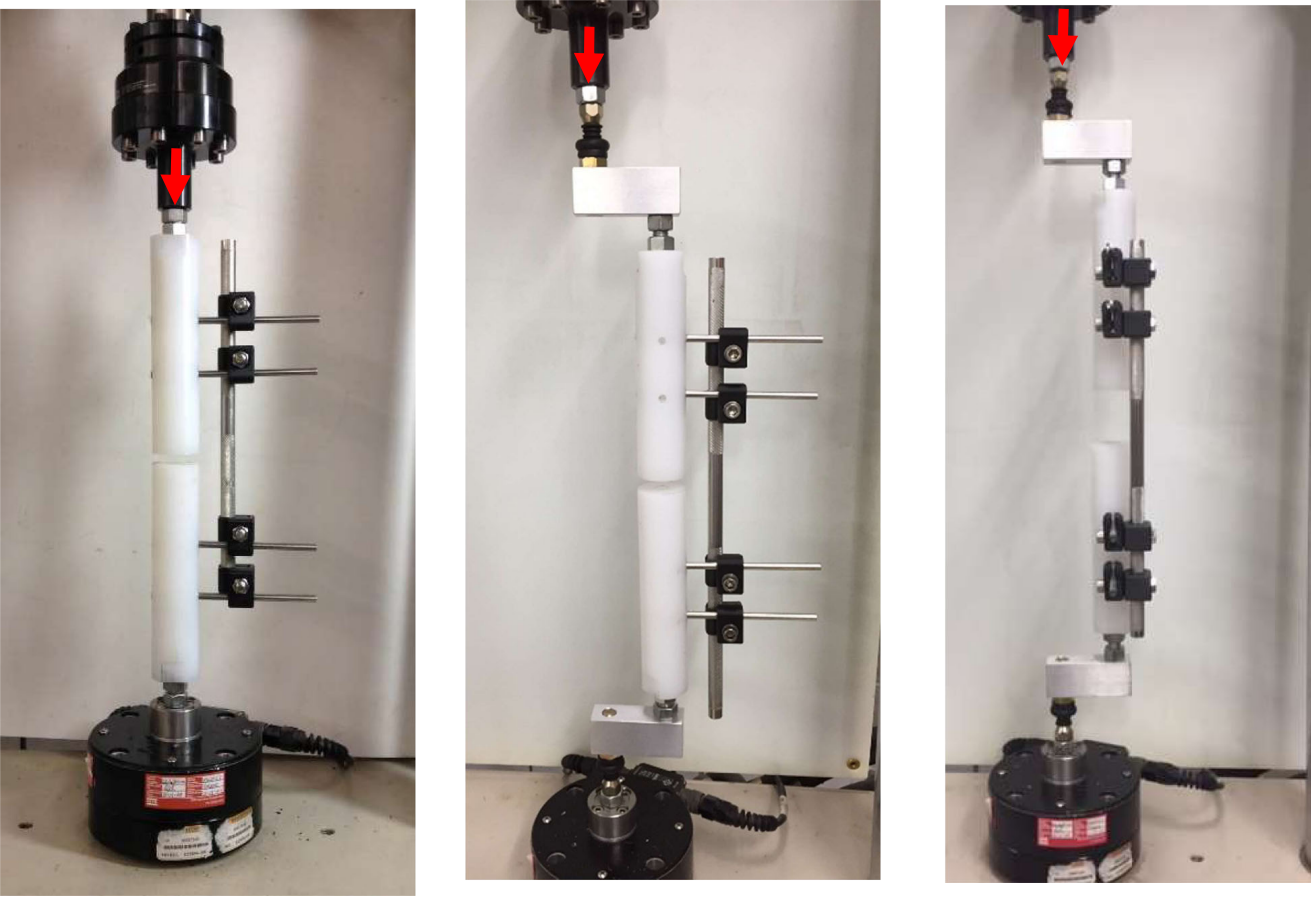
Left ro right: **a** Axial compression, **b** Over ball-and socket, AP-bending test set up providing an offset of 50 mm to the axial force **c** Over ball-and-socket, ML-bending test set up with a perpendicular force transmision.The direction of input force is denoted by the red arrow shown

This study included 3 different testing configurations: axial compression, anterior-posterior (AP) bending, and medial-lateral (ML) bending. Each test set up can be seen in Fig. 4. An input load was applied at a rate of 20 N/s, starting at 0 N using MTS tester Model 359 with hydraulic system MTS 505.11. There was a load limit of 750 N, or until there was a maximum vertical deformation of 6 mm. Axial transducer MTS Model 662.20C-01, calibrated, measured the deformation. Using MTS FlexTest software, force and deformation data was collected at a sampling rate of 30 Hz.

For axial compression, the input load was applied at the top of the UHWP rod fixture (Fig. 4a). AP bending underwent parallel force transmission as the input load force was offset from the UHWP rod axis by a 50.0 mm aluminum connecting part (Fig. 4b). ML bending underwent perpendicular force transmission as the input load force was offset from the UHWP rod axis by a 50.0 mm aluminum connecting part (Fig. 4c). In all testing configurations, the screws and nuts that joined the connecting and holding clamps were tightened at a standard torque of 18 N/m using a calibrated torque-measuring wrench by CDI Torque Products.

Six tests were performed for each biomechanical assessment. The load and displacement data were collected for each test and plotted on a load versus displacement graph. Prior to each test, 5 rounds of pre-conditioning loading were conducted on the linear external fixator per guidance in ASTM standard F1541. A new fixator was used for each test.

Biomechanical properties were characterized from the load versus displacement curves: the rigidity, safe load and yield load. Rigidity was defined as the slope of the linear portion of the curve [7]. Safe load was the load at which the deformation exceeded 1 mm. The length of 1 mm was chosen as the allowable separation for fractured bone in clinical settings to initiate osteosynthesis [11–14]. Yield load was defined as the load at which the curve deviates from its original linearity. ASTM standard F1541 was used to define the yield load, with an arbitrary offset of 0.1 mm. In each testing case, the rigidity was determined in a way such that any failure mode was avoided. Due to this, there was purposely no mode of failure and therefore ultimate strength was not determined.

## Results

The following dimensions of the clamps were assessed pre and post autoclave: the diameter where the rod interfaces with the clamp, clamp width, clamp height and clamp length. These values can be seen in Table 1. Dimensions of the 3D printed clamps after a full autoclave cycle shrunk 2.6%, 0.2%, 1.7% and 0.3%, respectively. While this does not affect the overall use of the clamps, it should be taken into consideration during manufacturing. All parts were found to be within the +/− 0.125 mm compliance from nominal value on all checked dimensions before and after autoclave.

**Table 1:** Key dimensions of the 3D printed clamp pre and post autoclave with standard deviations

	Diameter (mm)	Width (mm)	Height (mm)	Length (mm)
Pre Autoclave	12.457 +/− 0.037	24.068 +/− 0.046	21.928 +/− 0.049	31.190 +/− 0.068
Post Autoclave	12.127 +/− 0.059	24.030 +/− 0.040	21.563 +/− 0.041	31.103 +/− 0.123

**Fig. 5:**
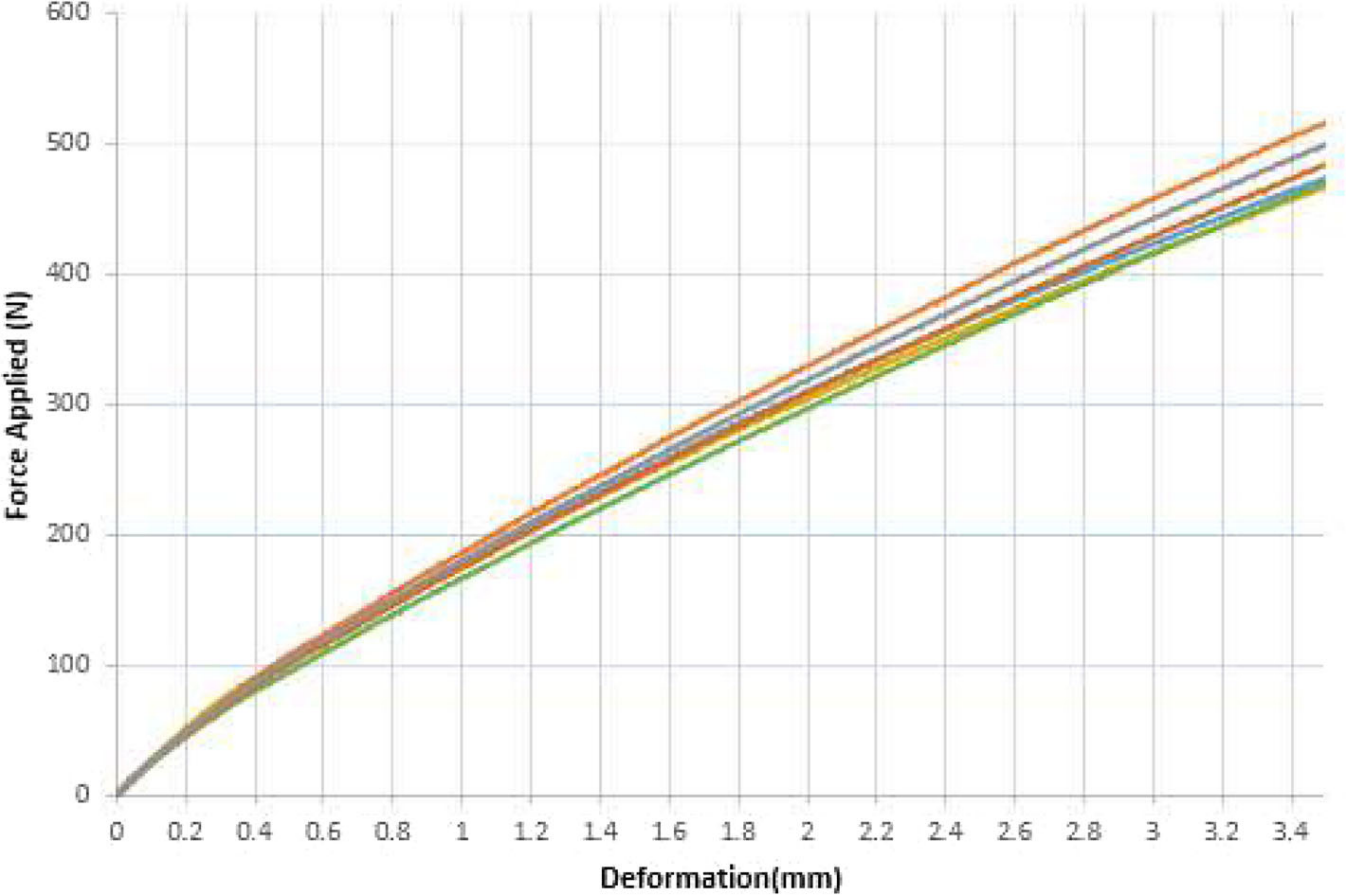
Axial compression on six evaluations (color coded)

**Fig. 6:**
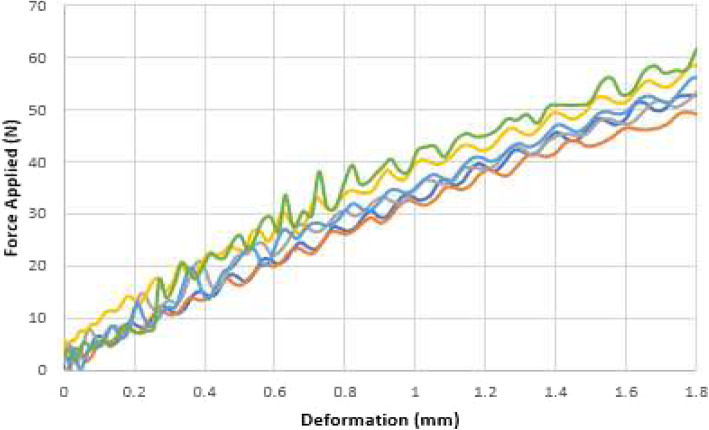
AP bending on six evaluations (color coded)

**Fig. 7:**
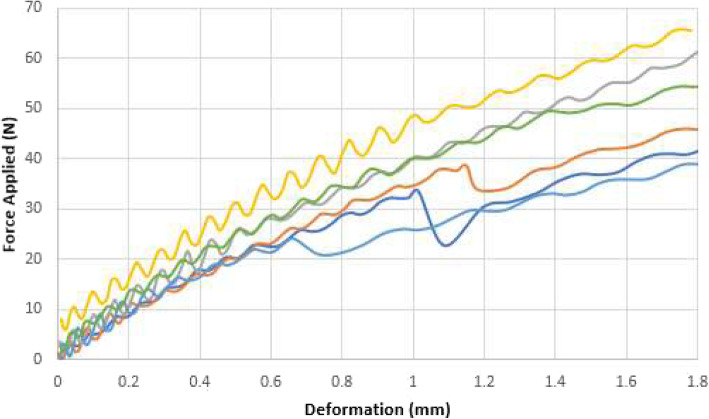
ML bending on six evaluations (color coded)

The displacement vs. the force applied for axial compression, AP-bending and ML-bending can be seen in Figs. 5, 6 and 7.

The mean rigidity results for axial compression, AP bending and ML Bending can be seen in Table 2. The average safe load determined from axial compression was 177.14 +/− 5.46 N. The average yield load was 122.92 +/− 4.47 N. Both were determined from the axial compression tests.

**Table 2:** Mean rigidity and standard deviation results determined from axial compression, AP bending and ML bending for the novel unilateral fixator

Axial compression (N/mm)	AP bending (N/mm)	ML bending (N/mm)
246.12 +/− 8.87	35.98 +/− 2.11	39.60 +/− 2.60-

## Discussion

The biomechanical properties of an external fixator closely correlate with its ability to properly facilitate bone healing. For example, it is necessary that an external fixator provides appropriate rigidity to the bone in order to allow only small interfragmentary movements during healing [15]. While the biomechanical properties of any external fixator do not prove that it will allow seamless healing, it is necessary to assess these properties before moving the fixator to a clinical setting.

Overall, the biomechanical properties of this novel fixator performed similarly compared to other external fixators that are currently used clinically. Specifically, the rigidity performance in the axial, AP, and ML directions is comparable with other studies that assess biomechanical performance of clinically used unilateral fixators [16].

The axial rigidity in [9] was determined in a similar methodological manner as this current study. Howmedica’s (currently Stryker) stacked half-frames Hoffman external fixation system (HF2) and Synthes’ (currently J&J) AO stacked half frames Hoffman external fixation system (AO HF2) are compared. The axial rigidity of the AO HF 2 frame was determined to be 2.58 +/− 0.03 kN/cm − 1 (258 +/− 30 N/mm). The axial rigidity of the Hoffmann HF2 was determined to be 1.5 +/− 0.04kN/cm (150 +/− 40 N/mm) [9]. Overall, the axial rigidity from the novel external fixator (246.12+/− 8.87 N/mm) is in the same range of the rigidity seen in the Howmedica’s and Synthes’ external fixators.

A study by R. M Sellei et al. assessed the ML and AP bending of 4 different Stryker’s Hoffman 3 external fixation configurations using ASTM standard F1541 testing. The results of these study are summarized in Table 3. The novel external fixator with 3D printed clamps had AP and ML bending rigidities of 35.98+/− 2.11 N/mm and 39.60+/− 2.60 N/mm, respectively. Its AP bending rigidity exceeds all AP bending rigidities from Sellei’s study [16]. Its ML bending rigidity exceeds that of both single rod configurations. The study’s results in comparison with the 3D printed fixator can be seen in Table 3.

**Table 3:** AP and ML bending rigidity values determined by R. M Sellei et al. for four different Hoffman configurations [16] with range reported in brackets. Compared with AP and ML bending rigidity values with associated standard deviations

Type of configuration	Double-rod configuration (N/mm)	Hybrid double rod configuration (N/mm)	Single rod direct link configuration (N/mm)	Single rod side arm configuration (N/mm)	3D printed single rod configuration (N/mm)
AP bending rigidity	29 [26–31]	17 [16–18]	29 [28–29]	13 [11–15]	35.98+/−2.11
ML bending rigidity	39 [38–39]	35 [34–37]	26 [25–27]	12 [10–13]	39.60+/−2.60

While it is necessary for an external fixator to be rigid, it is also beneficial in healing when bone micromovement is allowed [16]. But, any movement beyond a deformation of 1 mm is counter-productive [11–14]. The safe load of an external fixator is the load that causes a deformation of 1 mm, which is the maximum load that can be applied that still allows proper healing. The average assigned conventional weight for male patients is 70 kg (686 N) or 60 kg (588 N) for females [17, 18]. While the average safe load (177 N) of this fixator does not allow complete weight bearing of an average sized adult, it is sufficient for a non- full weight bearing patient undergoing fracture stabilization with soft tissue damage, which is about one quarter to one third of the average assigned conventional weight [14, 17].

The cost to produce this novel external fixator is below $150. If this novel external fixator is applied in a clinical setting after appropriate verification and validation techniques dictated by global or local regulatory health agencies, it would be readily accessible to the average patient. In a retrospective study, the cost associated with current definitive fixation for femoral shaft in a clinical setting was on average $15,374 [19]. Fixation devices used in other applications had a cost which ranged from $3556 to $20,486. An estimated manufacturing cost of $150, this before device verification and validation routines, is remarkably lower than the cheapest of any type of external fixation assessed in this study.

The assessment of the novel external fixator shows that its biomechanical properties are similar to current clinically used external fixators. Though further verification and validation routines remain to be conducted before certifying efficacy and safety of the novel fixator, it is reasonable to state that the design is easily reproducible. The components of this design that were not 3D printed (Schanz pins and microgroove stainless steel rod), are readily available worldwide, and the 3D printed clamps can be printed if an appropriate 3D printer and materials are purchased.

Specifically, the use of FDM 3D printed clamps composed of nylon with chopped carbon fiber provides a construction that supports axial, AP and ML compression. The material composition was also able to be sterilized by steam and still maintain its geometrical conformance. The shrinkage of the diameter, width, height, and length can be accounted for in clamp design.

A limitation of this study is the assessment of the biomechanical properties through torque applications. In the future, this assessment should be done per guidance in ASTM standard F1541 for torque tests. Also, this fixator should have been directly compared to commercially available linear external fixators. Collecting biomechanical data from an actual commercially available linear external fixator using the test set up of this study, all things equal, would have eliminated any study bias. Instead, data from previous studies were used for indirect comparison. This indirect comparison limits this study.

Additionally, the proof of using 3D printed parts to construct linear external fixators can be extrapolated on in the future by using the same fundamentals to design external fixators intended for other therapies beyond long bone fracture and soft tissue repair. The fundamentals could be extrapolated for limb lengthening and club foot treatment.

## Conclusion

Testing of the novel linear external fixator with 3D printed clamps per ASTM F1541 presented biomechanical properties that are comparable to those required in clinical settings. At an applied force of 180 Newtons, there was a deformation of 1 mm. On average, this is about one third of an average human’s body weight and sufficient for a non- full weight bearing patient undergoing fracture stabilization with soft tissue damage, which is the current form of treatment [17].

The utilization of FDM printing methodology using nylon and chopper carbon fiber as material for the clamp production was suitable for this application. This technique allowed for the clamps to provide support in order to produce appropriate axial compression, AP and ML rigidities. The material composition also allowed for the clamps to maintain the appropriate shape with minimal dimensional shrinkage that can be accounted for in clamp design.

Overall, the biomechanical properties of the novel fixator assessed in this study were comparable to external fixators that are currently being used in clinical settings. While the biomechanics were comparable, the low cost of this design, and the utilization of 3D printed clamps and additional readily available parts, meets the need for inexpensive, readily available external fixators in developing countries. However, before this novel fixator can be used in a clinical setting, further verification and validation techniques dictated by global or local regulatory health agencies to evaluate safety and effectives are needed.

## Data Availability

Data is available upon request.
